# ZFP580, a Novel Zinc-Finger Transcription Factor, Is Involved in Cardioprotection of Intermittent High-Altitude Hypoxia against Myocardial Ischemia-Reperfusion Injury

**DOI:** 10.1371/journal.pone.0094635

**Published:** 2014-04-10

**Authors:** Xiang-yan Meng, Hai-long Yu, Wen-cheng Zhang, Tian-hui Wang, Xia Mai, Hong-tao Liu, Rui-cheng Xu

**Affiliations:** 1 Institute of Health and Environmental Medicine, Academy of Military Medical Sciences, Tianjin, China; 2 Department of Physiology and Pathophysiology, Logistics University of Chinese People's Armed Police Force, Tianjin, China; 3 Tianjin Key Laboratory for Biomarkers of Occupational and Environmental Hazard, Logistics University of Chinese People's Armed Police Forces, Tianjin, China; Virginia Commonwealth University, United States of America

## Abstract

**Background:**

ZFP580 is a novel C2H2 type zinc-finger transcription factor recently identified by our laboratory. We previously showed that ZFP580 may be involved in cell survival and growth. The aim of this study was to elucidate whether ZFP580 is involved in the cardioprotective effects of intermittent high-altitude (IHA) hypoxia against myocardial ischemia-reperfusion (I/R) injury.

**Methods and Results:**

After rats were subjected to myocardial ischemia for 30 min followed by reperfusion, ZFP580 expression in the left ventricle was measured. ZFP580 protein expression was found to be up-regulated within 1 h and decreased at 2 h after reperfusion. Comparing normoxic and IHA hypoxia-adapted rats (5000 m, 6 h day^−1^, 6 weeks) following I/R injury (30 min ischemia and 2 h reperfusion), we found that adaptation to IHA hypoxia attenuated infarct size and plasma leakage of lactate dehydrogenase and creatine kinase-MB. In addition, ZFP580 expression in the myocardium was up-regulated by IHA hypoxia. Consistent with this result, ZFP580 expression was found to be significantly increased in cultured H9c2 myocardial cells in the hypoxic preconditioning group compared with those in the control group following simulated I/R injury (3 h simulated ischemic hypoxia and 2 h reoxygenation). To determine the role of ZFP580 in apoptosis, lentivirus-mediated gene transfection was performed in H9c2 cells 72 h prior to simulated I/R exposure. The results showed that ZFP580 overexpression significantly inhibited I/R-induced apoptosis and caspase-3 activation. H9c2 cells were pretreated with or without PD98059, an inhibitor of ERK1/2 phosphorylation, and Western blot results showed that PD98059 (10 µM) markedly suppressed I/R-induced up-regulation of ZFP580 expression.

**Conclusions:**

Our findings demonstrate that the cardioprotective effect of IHA hypoxia against I/R injury is mediated via ZFP580, a downstream target of ERK1/2 signaling with anti-apoptotic roles in myocardial cells.

## Introduction

Coronary artery disease (CAD) and acute myocardial infarction are two of the major causes of death worldwide. The high mortality rates associated with these diseases reflect a lack of effective strategies to reduce ischemia–reperfusion (I/R) injury. Therefore, developing alternative approaches for reducing post-ischemic injury based on thorough understanding of intrinsic cardioprotective mechanisms is very important. During myocardial ischemia or reperfusion, expression of transcription factors such as c-fos, c-jun, junB, and Egr-1 is up-regulated [1], [2]. A number of these proteins are also involved in endogenous cardioprotection against myocardial I/R injury. Recently, a novel gene, *ZNF580*, which is associated with low-density lipoprotein stimulation in human vascular endothelial cells, was cloned in our laboratory from the aortic tissue cDNA library by Zhang and colleagues [3]. Further bioinformatic analysis indicated that the *ZNF580* gene encodes a 172-amino acid polypeptide containing three repeat tandem C2H2-type zinc finger motifs at its carboxyl terminus. The fusion protein EGFP-ZNF580 is localized in the nuclei of MGC803 and HEK293 cell lines [4]. These data suggest that ZNF580 is a C2H2-type nuclear transcription factor. C2H2-zinc finger genes constitute the largest class of transcription factors within the human genome. These genes are generally involved in crucial cell functions, such as survival and growth [5]. Northern blot analysis of multiple organs revealed that *ZNF580* is ubiquitously expressed in human tissues and shows the highest expression in the heart [3]. In addition, ZNF580 is vital in the migration and proliferation of vascular endothelial cells [6]. Therefore, we hypothesized that ZNF580 might be involved in cardiovascular diseases and could be used as a new molecular target for treating such diseases. The murine homologue of *ZNF580*, namely, *ZFP580*, was subsequently cloned in our laboratory and investigated in rats [7]. Our previous study showed that ZFP580 expression changes during myocardial I/R and can be up-regulated by L-arginine preconditioning [8]. However, the exact role of ZFP580 in myocardial I/R remains unclear.

Adaptation to high-altitude hypoxia confers long-lasting cardiac protection against acute I/R injury. This phenomenon has been demonstrated in both human populations living at high altitudes and in several animal studies [9]. Intermittent high-altitude (IHA) hypoxia induces a wide variety of myocardial adaptive changes that can be considered protective [10]. Activation of the prosurvival extracellular signal-regulated kinase 1 and 2 (ERK1/2) pathway has been demonstrated to confer cardioprotection [11]. Additionally, IHA hypoxia has been suggested to reduce myocardial infarct size through ERK pathway activation [10]. Given that ZFP580 is mainly expressed in the heart and may be involved in cell survival and maintenance of normal cellular function, we hypothesized that ZFP580 might be involved in the cardioprotective effects of IHA hypoxia against myocardial I/R injury via ERK1/2 activation.

## Materials and Methods

### Ethics statement

All animals used in this study received humane care in compliance with the Guide for the Care and Use of Laboratory Animals published by the US National Institutes of Health (NIH, publication number 85–23, revised 1996, http://grants.nih.gov/grants/olaw/olaw.htm), and all the protocols were approved by the Animal Subjects Committee of the Academy of Military Medical Sciences, Beijing, China.

### Animals

Male Wistar Rats weighing 150 g to 180 g were obtained from the Animal Center of the Academy of Military Medical Sciences (Beijing, China). The rats were maintained under standard conditions (ambient temperature 21–23°C, with a 12 h dark-light cycle) with ad libitum access to food and tap water.

### IHA hypoxia

In accordance with the method of Chen et al. [12], the rats were exposed to IHA hypoxia in a hypobaric chamber (equivalent to an altitude of 5000 m, with a barometric pressure of 420 mmHg) for a 6 h period each day for 42 d. The rat body weights during this period increased from 150 g–180 g to 380 g–400 g. Age-matched rats were maintained in normoxic environment for 42 d. The body weights and weight gains of these normoxic rats were identical to those of rats exposed to IHA hypoxia. All animals had free access to water and standard laboratory diet.

### I/R in Rat Hearts

IHA hypoxic and age-matched normoxic rats were anesthetized with 25% ethylurethanm solution (0.4 mL/100 g body weight, ip). Tracheotomy was performed on the animals, after which an intubation cannula was connected to a rodent ventilator. The breathing rate was set to 80 breaths/min, and the tidal volume was 1.0 mL/100 g body weight. The heart was exposed, and the left coronary artery (LCA) was temporarily ligated with a 6–0 silk suture. During this operation, a piece of lightweight rubber rod (2 mm diameter, 1 cm length) was placed between the LCA and the 6–0 suture. After 30 minutes of LCA occlusion, reperfusion was initiated by releasing the ligature and removing the rubber rod. The loosened suture was left in place to help identify the ischemic area of the left ventricle.

### Assessment of Myocardial Infarct Size

At the end of the experiment, the LCA was religated at the original site, and the area at risk (AAR) was determined by injecting 1 mL of 4% phthalocyanine blue dye via the right common carotid artery. The heart was removed and frozen at −20°C for 1 h. The left ventricle was cut into five to six transverse slices of 1.5 mm–2 mm thickness. The slices were then incubated at 37°C in a 1% solution of buffered 2,3,5-triphenyltetrazolium chloride (TTC) (pH 7.4) for 20 min to identify the area of necrosis (AN) within the AAR. The AN (pale), AAR (bright red), and non-ischemic portion of the heart specimens (purple) were photographed and measured. The size and necrosis percentage of the AAR were calculated in each individual slice by planimetry (Image-Pro Plus software).

### Determination of lactate dehydrogenase (LDH) activity and creatine kinase cardiac isoenzyme (CK–MB) concentration

To evaluate myocardial necrosis, plasma was collected and CK–MB and LDH, which are two biochemical markers of cellular necrosis, were measured at 0 min, 15 min, 30 min, 1 h, 2 h and 4 h after reperfusion. The CK–MB levels were determined using a commercially available rat ELISA kit (R&D Systems), whereas the LDH activity was determined using a biochemistry detection kit (Jian–Cheng Biotechnology Company, Nanjing, China).

### Cell culture and experimental protocols

An embryonic rat heart-derived cell line, H9c2, was obtained from the American Type Culture Collection (Manassas, VA, USA). Cells were cultured in Dulbecco's modified Eagle's medium (DMEM) supplemented with 10% fetal bovine serum (Gibco, Australia) and 1% penicillin–streptomycin.

The general experimental protocols are as follows:

- Group I (control): The H9c2 cells were incubated in serum-free DMEM during the entire experimental period;
- Group II (simulated I/R, SI/R): SI/R was induced by placing cells in an anoxia chamber and replacing the medium with an ischemic buffer containing the following (in mmol/L, pH 6.2): 1.0 KH_2_PO_4_, 10.0 NaHCO_3_, 1.2 MgCl_2_·6H_2_O, 25.0 HEPES, 74.0 NaCl, 16.0 KCl, 1.2 CaCl_2_, and 20.0 Na lactate, which was previously equilibrated in the anoxia chamber (95% N_2_, 5% CO_2_, 37°C). After incubation under ischemic hypoxic conditions for 3 h, the cells were incubated in serum-free DMEM under normoxic conditions (20% O_2_, 5% CO_2_) at 37°C for the indicated periods; and
- Group III (hypoxic preconditioning, HPC): Cells were exposed to HPC prior to SI/R. HPC was induced by exposing the H9c2 cells to 10 min of hypoxia and 20 min of reoxygenation for 3 cycles.

### Cell viability assessment

Cell viability was assessed using a [3-(4, 5-dimethylthiazolyl-2)-2,5-diphenyltetrazolium bromide] (MTT) cell proliferation assay kit. In brief, the medium was replaced, and cells were incubated for an additional 4 h prior to assessment of cell viability using MTT staining. Optical density was assessed at 490 nm using a microplate reader (Bio-Rad Laboratories Inc., USA). Cell viability was expressed as the percentage of absorbance from treated cells compared to untreated cells.

### Lentivirus-mediated gene transfection

Lentiviral vectors expressing ZFP580 full-length genes (lenti-ZFP580), or siRNA directed against ZFP580 (lenti-siRNA), or negative control (lenti-NC) were constructed by GenePharma (Shanghai GenePharma Co., China). Lentivirus-mediated gene transfection was performed by adding a minimal volume of 10% fetal bovine serum DMEM containing lenti-ZFP580, or lenti-siRNA, or lenti-NC to the dish at a multiplicity of infection (MOI, which is the ratio of infectious virus particles to the number of cells being infected) value of 200. All experiments were performed in H9c2 cells 72 h after lentivirus-mediated gene transfection.

### ERK1/2 blocking assay

To evaluate the role of ERK1/2 activation in ZFP580 expression, a selective inhibitor of ERK1/2 phosphorylation, PD98059 (Sigma, USA), was added to the medium at a final concentration of 10 µM 1 h prior to the experimental treatment.

### Quantitative real-time RT-PCR assay

Total RNA was extracted from ischemic left ventricular tissues or H9c2 cells with the TRIzol reagent (Invitrogen, USA) and then reverse-transcribed into cDNA. The synthesized cDNA was amplified using Platinum Taq PCRx DNA polymerase (Transgen, China). The specific primers used for ZFP580 were as follows: ZFP580 forward 5′-ACATCATTTCGTCTTTTCTTCTG-3′ and ZFP580 reverse 5′-GGTGCTTTTGTCATTTCTTCCAC-3′. The PCR conditions for ZFP580 were 15 s at 95°C, 34 s at 63°C, and 45 s at 72°C for 40 cycles. Expression of glyceraldehyde-3-phosphate dehydrogenase (GAPDH), a housekeeping gene, was used as an internal control. The results were analyzed using the Applied Biosystems 7500 system v1.4.0 software.

### Western blotting

Frozen ischemic left ventricular tissues or cells samples were homogenized in a Radio-Immunoprecipitation Assay (RIPA) lysis buffer. For direct immunoblotting, aliquots of the lysate were mixed with 5× SDS-PAGE sample loading buffer (containing 5% 2-mercaptoethanol) and boiled for 10 min. For each sample, 60 µg of protein was loaded into an acrylamide gel (12% and 15% gels were used) and subsequently transferred onto a polyvinylidene fluoride membrane (Millipore, 0.22 µm). The membranes were blocked in 5% (w/v) nonfat dry milk in Tris-buffered saline containing Tween 20 for 1 h at room temperature and then incubated with primary antibodies overnight at 4°C. After washing, the membrane strips were incubated with anti-rabbit or anti-mouse IgG (Cell Signaling Technology, Inc. MA, USA) conjugated to horseradish peroxidase (HRP) for 1 h. Protein bands were detected by the standard enhanced chemiluminescence (ECL) method, and images were digitized and relative band intensities were measured by densitometry using Image Lab software version 4.1.0 (Bio-Rad).

### Apoptosis assay by flow cytometry

Flow cytometry was performed with 7-aminoactinomycin (7-AAD) and phycoerythrin (PE)-labeled annexin V to detect phosphatidylserine externalization as an endpoint indicator of early apoptosis following the manufacturer's instructions. In brief, cells (1×10^6^/well) were subjected to simulated I/R 72 h after lentivirus-mediated gene transfection and were then detached from each dish by incubation with trypsin (0.25 mg/mL). The cells were washed with phosphate-buffered saline and then stained with 500 µL of a labeling solution containing PE-labeled annexin V and 7-AAD for 15 min at room temperature. Subsequently, the samples were analyzed using an Epics Elite flow cytometer, and the data were examined using the Modfit 3.0 software. During the early stages of apoptosis, cells display phosphatidylserine on their outer cell membranes, which is readily detected by annexin V. During the later stages of apoptosis, the plasma membrane becomes increasingly permeable, and 7-AAD can move across the cell membrane to bind to cellular DNA. Therefore, 7-AAD is useful in identifying cells that have lost membrane integrity as a result of necrosis. In our study, cells in the early stages of apoptosis were found to be annexin V-positive and 7-AAD-negative, whereas those in late-stage apoptosis were positive for both annexin V- and 7-AAD.

### Statistical analysis

Data are expressed as the mean ± standard error of the mean (SEM). Statistically meaningful differences were determined using a two-tailed student's *t* test or ANOVA. A p-value <0.05 was considered statistically significant.

## Results

### Cardioprotection of IHA hypoxia against myocardial I/R injury

**Figure 1:**
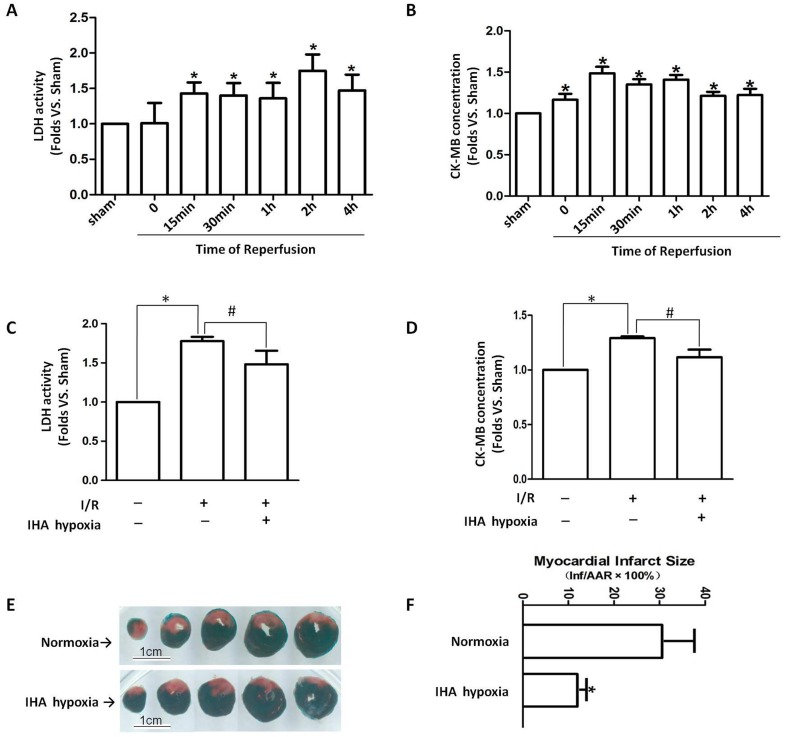
Cardioprotective effects of IHA hypoxia against myocardial I/R injury. After rats were subjected to 30(A) LDH activity and (B) CK–MB concentration in the plasma were measured. Following 30 min of myocardial ischemia and 2 h of reperfusion, adaptation to IHA hypoxia attenuated I/R-induced (C) LDH and (D) CK–MB plasma leakage compared with the normoxia group. (E) Representative images of rat heart slices stained with 10% TTC in which infarct areas are pale, viable tissues are red, and non-ischemic portions of the heart specimens are purple. (F) Graphs show that I/R-induced cardiac infarction was attenuated by IHA hypoxia adaptation. n = 6–8 rats per group.*p<0.05 vs. sham group, #p<0.05 vs. I/R group in normoxia. Values are presented as mean ± SEM.

LDH and CK–MB plasma leakage significantly increased during reperfusion. The increase in LDH activity delayed the appearance of the maximum CK–MB content (Figs. 1A and 1B). This result indicates that reperfusion caused further damage after myocardial ischemia. However, after 30 min of myocardial ischemia and 2 h of reperfusion, adaptation to IHA hypoxia suppressed the I/R-induced LDH and CK–MB plasma leakage (Figs. 1C and 1D). In addition, I/R-induced cardiac infarction was evaluated using TTC staining. Representative sections of left ventricle (Fig. 1E) showed that I/R-induced infarction (pale area) was attenuated in the IHA hypoxia group (Fig. 1F).

### Involvement of ZFP580 in the cardioprotection against myocardial I/R injury caused by IHA hypoxia

**Figure 2:**
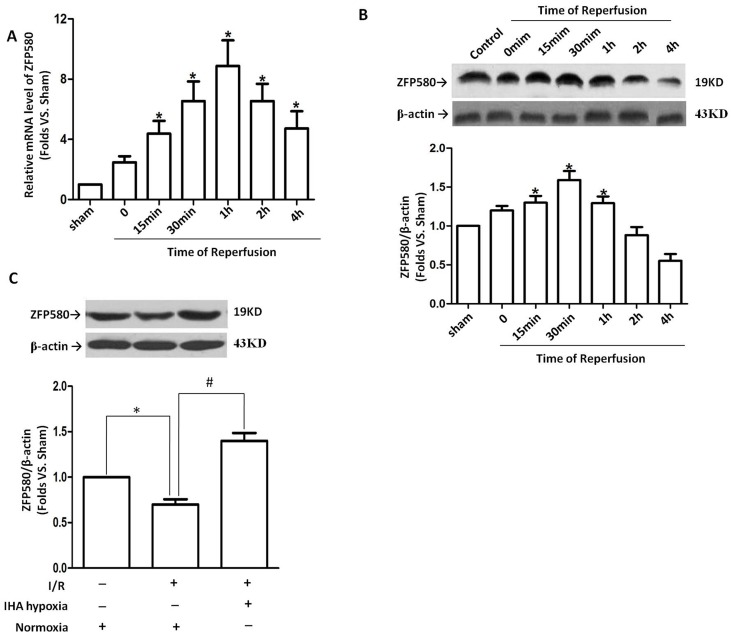
IHA hypoxia induced ZFP580 expression in the myocardium. Following 30(A) and protein (B) levels in the myocardium were assayed at different time points after reperfusion. (C) After 30 min of myocardial ischemia and 2 h of reperfusion, ZFP580 protein levels in IHA hypoxia groups were markedly higher than those in normoxia groups. n = 6–8 rats per group.*p<0.05 vs. sham group, #p<0.05 vs. I/R group in normoxia. Values are presented as mean ± SEM.

After 30 min of myocardial ischemia, ZFP580 mRNA and protein levels in the left ventricle (LV) were monitored from 15 min to 4 h after reperfusion. I/R induced significant ZFP580 mRNA expression at all time points, with the maximum effect observed at 1 h after reperfusion (Fig. 2A). ZFP580 protein level was increased compared with that in the sham group with peak level occurring at 30 min after reperfusion and the overall elevation lasting for 1 hr after reperfusion (Fig. 2B). These early increases disappeared and ZFP580 protein levels returned to control levels 2 h after reperfusion. However, after 2 h of reperfusion, rats adapted to IHA hypoxia maintained a higher level of ZFP580 protein in the ischemic LV compared with those in the normoxia group (Fig. 2C).

**Figure 3:**
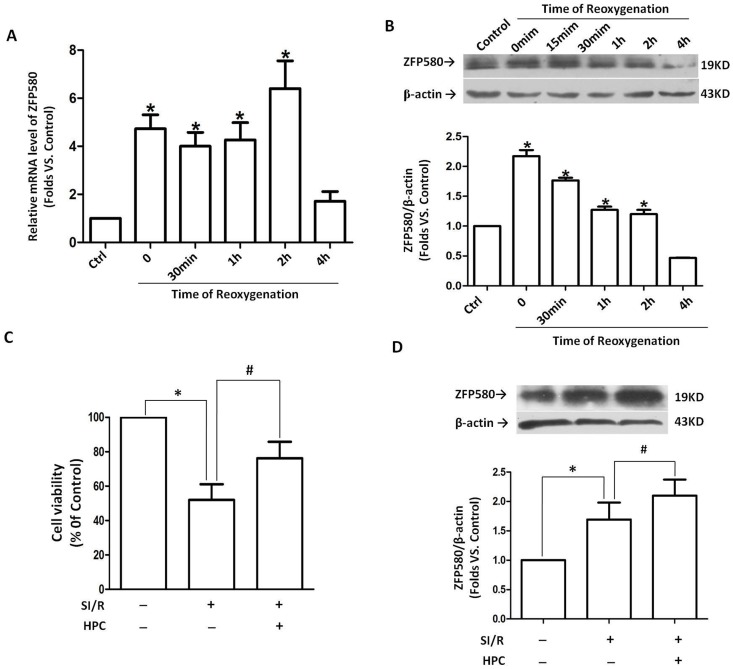
ZFP580 expression in H9c2 cells was induced by SI/R or HPC. H9c2 cells were subjected to 3(A) and protein (B) levels were assayed by quantitative real-time PCR and western blot assays. (C) MTT staining results showed HPC cytoprotection against SI/R-induced cell death. (D) Western blot results showed HPC-induced up-regulation of ZFP580 expression in H9c2 cells. HPC was induced by exposing H9c2 cells to 10 min of hypoxia and 20 min of reoxygenation for 3 cycles before subjecting them to SI/R. Three experiments were performed for each group and each experiment was replicated twice. Values are presented as mean ± SEM. *p<0.05 vs. control, #p<0.05 vs. SI/R group.

To further investigate the role of ZFP580 in protection against I/R injury caused by IHA hypoxia, we established SI/R and HPC models in H9c2 myocardial cells. ZFP580 mRNA and protein expression were found to have significantly increased in H9c2 myocardial cells subjected to SI/R. This result is similar to those obtained from animal experiments. ZFP580 mRNA expression significantly increased after simulated ischemic hypoxia treatment and remained at a high level within 2 h after reoxygenation (Fig. 3A). Furthermore, increases in ZFP580 protein level were first observed after simulated ischemic hypoxia and remained at a high level within 2 h after reoxygenation (Fig. 3B). Meanwhile, MTT staining results showed evidence of HPC cytoprotection against SI/R-induced cell death in H9c2 cells (Fig. 3C). ZFP580 expression significantly increased in the HPC group compared with those in the control and SI/R groups (Fig. 3D). Taken together, these results indicate that HPC increased ZFP580 protein levels in H9c2 cells and conferred protection against SI/R.

**Figure 4:**
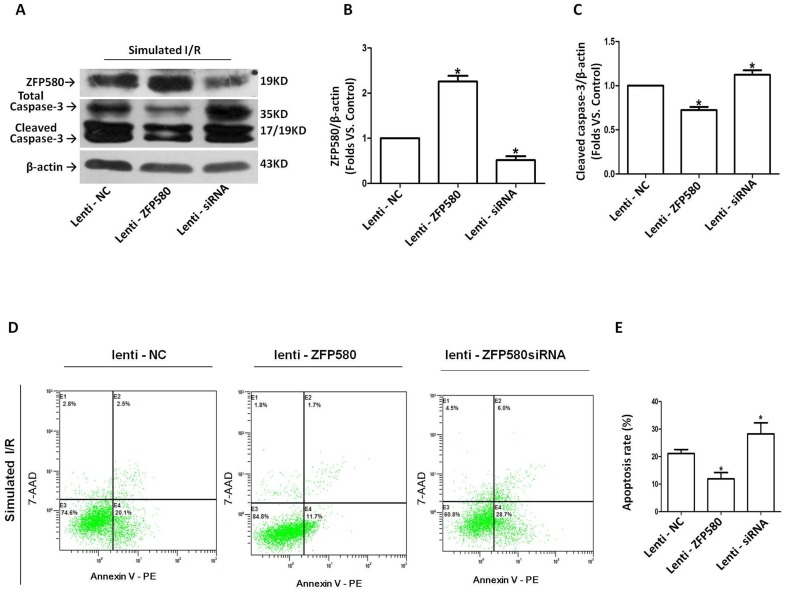
ZFP580 overexpression in H9c2 cells inhibited SI/R-induced apoptosis and caspase-3 activation. H9C2 cells were transfected with lentiviral vectors expressing full-length ZFP580 (lenti-ZFP580), siRNA directed against ZFP580 (lenti-siRNA) or negative control (lenti-NC) for 72 hours, then underwent SI/R. (A–C): Western blot analysis revealed significant effects of ZFP580 overexpression on cleavage of caspase-3. H9c2 cells (1×10^6^) were collected and stained with Annexin V-PE/7-AAD, and quantified by flow cytometry. (D) Dot plots showing Annexin-V-PE/7-AAD staining of H9c2 cells examined by flow cytometry. (E) Percentage of apoptotic cells as measured by Annexin-V staining in each of the indicated treatments. Three experiments were performed for each group and each experiment was replicated twice. Values are presented as mean ± SEM. *p<0.05 vs. NC.

In order to determine the role of ZFP580 in I/R, lentivirus-mediated transfection was performed on H9c2 myocardial cells 72 h prior to SI/R exposure. Validation of ZFP580 expression upon transfection was done by Western blot analysis of cell lysates (Figs. 4A and 4B). Examination of cleaved caspase-3 expression in H9c2 cells showed that ZFP580 overexpression prevented caspase-3 activation (Fig. 4C). To further determine whether change in ZFP580 expression affected myocardial apoptosis, the degree of SI/R-induced apoptosis was determined by annexin V/7-AAD staining and flow cytometry analysis. Results showed that ZFP580 overexpression significantly inhibited apoptosis of H9c2 cells, whereas suppression of ZFP580 expression increased their apoptotic rates compared with the negative controls (Figs. 4D and 4E).

### Association of ERK1/2 activation with ZFP580 up-regulation

**Figure 5:**
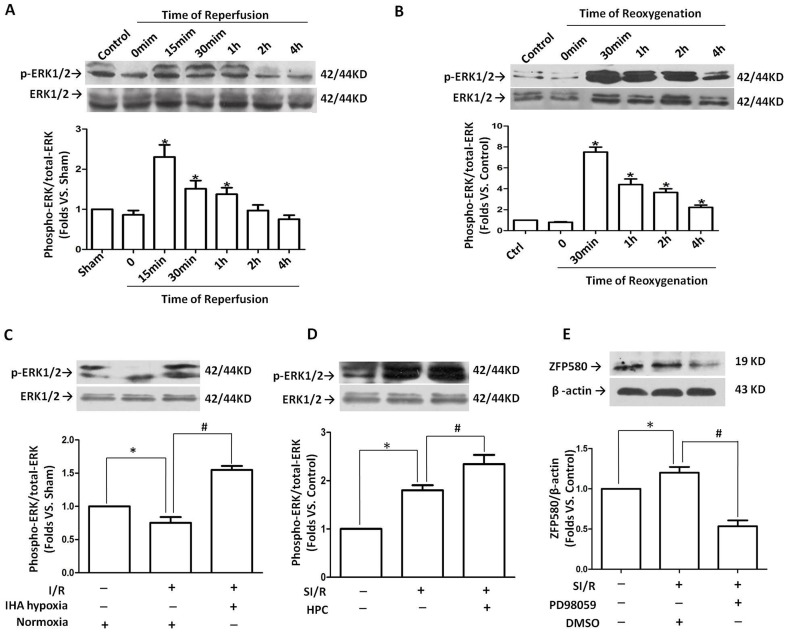
Association of ZFP580 up-regulation with ERK1/2 activation induced by I/R. (A) ERK1/2 phosphorylation levels in hearts subjected to I/R. (B) ERK1/2 phosphorylation levels in H9c2 cells subjected to SI/R. Both IHA hypoxia (C) and HPC (D) increased ERK1/2 activation in the myocardium and in H9c2 myocardial cells subjected to I/R. Western blot results are expressed as the ratio of phosphorylated ERK1/2 relative to total ERK1/2. (E) Treatment with PD98059, a selective inhibitor of ERK1/2 phosphorylation, significantly suppressed SI/R-induced ZFP580 expression in H9c2 myocardial cells. A total of 6–8 rats were used per group for *in vivo* experiments, and *in vitro* experiments were repeated three times. Values are presented as mean ± SEM. *p<0.05 vs. control, #p<0.05 vs. I/R group.

To elucidate the molecular mechanism of ZFP580 involvement in cardioprotection against I/R injury, the prosurvival ERK1/2 signaling pathway was examined. As shown in Figure 5A, rapid and transient activation of ERK1/2 was observed within 2 h after reperfusion, and the peak values appeared 15 min after reperfusion. In H9c2 cells subjected to SI/R, similar activation of ERK1/2 occurred after 30 min of reoxygenation and remained at a high level up to 4 h after reoxygenation (Fig. 5B). In addition, *in vivo* and *in vitro* experiments confirmed that both IHA hypoxia and HPC could increase ERK1/2 activation in I/R-exposed myocardial cells (Figs. 5C and 5D).

Interestingly, the observed increases in ERK1/2 activation were associated with parallel increases in ZFP580 protein levels. To determine whether the ERK1/2 signaling pathway is involved in the I/R-induced up-regulation of ZFP580 expression, H9c2 cells were pretreated with or without PD98059, an inhibitor of ERK1/2 phosphorylation, and ERK1/2 phosphorylation and ZFP580 protein levels in cell lysates were determined. As shown in Figure 5E, PD98059 treatment (10 µM) significantly suppressed the I/R-induced up-regulation of ZFP580 expression at the protein level (p<0.05).

## Discussion

Adaptation to chronic hypoxia confers long-lasting cardiac protection against acute I/R injury. This phenomenon has been demonstrated both in human populations living at high altitudes and in numerous animal studies. In this study, we confirmed that adaptation of rats to IHA hypoxia attenuated infarct size and increased the tolerance of their hearts to acute I/R injury. Furthermore, HPC exhibited cytoprotection against simulated I/R in H9c2 myocardial cells. These results are consistent with those of several previous studies [13]–[15]. The precise mechanism by which IHA hypoxia has protective effects against myocardial I/R injury is not clear. Several studies have reported that the protective effects of long-term chronic hypoxia as well as short-lived HPC, including both early and delayed forms, against acute I/R injury are related to the expression and/or activation of regulatory proteins [16]–[18]. In this study, we found that ZFP580 mRNA and protein expression levels in ischemic myocardium or H9c2 myocardial cells were significantly increased by I/R and remained at high levels during the early-phase of reperfusion (within 2 h after reperfusion or reoxygenation). Moreover, IHA hypoxia or HPC led to high levels of ZFP580 protein that lasted up to 2 h after reperfusion. These results suggest that ZFP580 may be a stress-related protein that is expressed at the early phase of myocardial I/R and has protective effects on myocardial cell survival. In addition, we have observed a more obvious increase in the mRNA expression of ZFP580 than the corresponding protein level. We think a possible reason for the discrepancy is that they are under different modes of transcriptional and translational controls. Based on a bioinformatic analysis, we found that the 3′ untranslated region of ZFP580 mRNA may be regulated by miR-1 and miR-206. Shan, et al [19] reported that expression of miR-1/miR-206 was up-regulated in a rat model of myocardial infarction. Therefore, it is possible that miR-1 or miR-206 or both are up-regulated during I/R, and this result in the tight regulation of ZFP580 mRNA. We are currently conducting experiments to test this hypothesis in the lab.

The two major forms of cell death implicated in post-reperfusion tissue injury are necrosis and apoptosis. Apoptosis is a regulated process that results from the pathology of myocardial infarction [20]. Several studies have suggested that targeting the reperfusion-induced apoptotic component of cell death can impact both the apoptotic and necrotic components of cell death, and can result in reduction in infarct size and improved contractile function [21]. In this study, we found that lentivirus-mediated overexpression of ZFP580 in H9c2 cells attenuated I/R-induced apoptosis as well as activation of caspase-3, which is a crucial protein in the apoptotic cascade. However, suppression of ZFP580 expression by RNAi enhanced I/R-induced cell apoptosis and caspase-3 activation. These findings suggest that endogenous ZFP580 is a component of the anti-apoptotic machinery within cardiomyocytes, and that it is involved in cardioprotection against myocardial I/R injury. However, elucidation of the specific mechanism underlying the anti-apoptotic effect of ZFP580 and the signaling pathways involved requires additional investigation.

Previous studies have shown that a multitude of intracellular signal transduction pathways are activated during myocardial I/R injury. Among them, mitogen-activated protein kinases (MAPKs) are key regulators of cell function and survival [22]. ERK1/2 is an important subfamily of MAPKs that control a broad range of cellular activities and physiological processes. Several studies have provided evidence supporting a protective role of the ERK1/2 signaling pathway against I/R injury [23], [24]. Evidence showing anti-apoptotic effects of ERK1/2 signaling on cardiomyocytes during reperfusion is also known [25]. Accordingly, the ERK1/2 signaling pathway has been identified as a central component of the so-called “Reperfusion Injury Salvage Kinase” (RISK) pathway [26]. ERK1/2 acts as a messenger between the cytoplasm and the nucleus and possibly protects the myocardium against reperfusion injury via activation or phosphorylation of downstream target molecules comprising mainly of transcription factors. ERK1/2 is also implicated in cellular survival, which it mediates by recruiting anti-apoptotic pathways [27].Based on these facts, we hypothesized that ZFP580 is a transcription factor regulated by ERK1/2 activation and mediates the anti-apoptotic effects of the ERK1/2 signaling pathway. Generally, transient ERK1/2 activation can regulate the activity of survival and apoptotic signaling molecules, prolonged activation of ERK1/2 can up- or down-regulate the expression of such molecules in a way that has anti-apoptotic effects [28]. The consequences of transient or prolonged activation of ERK1/2 can be different depending on the downstream effectors involved. In this study, ERK1/2 could be transiently activated by I/R or SI/R, but IHA hypoxia caused prolonged activation of ERK1/2 (ERK1/2 phosphorylation was observed after 42 d of chronic intermittent hypoxia). Regardless of whether ERK1/2 underwent transient or prolonged activation, changes in ERK1/2 phosphorylation were associated with parallel changes in ZFP580 protein levels. In addition, treatment with 10 µM PD98059, a known inhibitor of ERK1/2 phosphorylation, markedly attenuated I/R-induced up-regulation of ZFP580 expression in H9c2 myocardial cells. Thus, our results suggest that ERK1/2 activation may be involved in the up-regulation of ZFP580 expression, and this may underlie the anti-apoptotic effect of the ERK signaling pathway.

To the best of our knowledge, this is the first report to provide experimental evidence of anti-apoptotic effect of ZFP580 following I/R injury. Both IHA hypoxia and HPC could up-regulate ZFP580 expression, suggesting that ZFP580 may be a possible therapeutic target for treating myocardial injury. Moreover, this study provides evidence that I/R-induced ZFP580 up-regulation depends partly on transient or prolonged activation of ERK1/2. Therefore, ZFP580 may be involved in the RISK pathway, of which ERK1/2 is a major component, in the heart. The results of this study may be useful in developing novel strategies that target ZFP580 to protect the hearts of CAD patients.
